# Benefits of starting hypothermia treatment within 6 h vs. 6–12 h in newborns with moderate neonatal hypoxic-ischemic encephalopathy

**DOI:** 10.1186/s12887-018-1013-2

**Published:** 2018-02-12

**Authors:** Wen Jia, Xiaoping Lei, Wenbin Dong, Qingping Li

**Affiliations:** Department of Neonatology, The Affiliated Hospital of Southwest Medical University, Luzhou, 646000 China

**Keywords:** Hypoxic ischemic encephalopathy, Mild hypothermia, Time window

## Abstract

**Background:**

It has been suggested that mild hypothermia treatment of hypoxia-ischemic encephalopathy (HIE) should start within 6 h after HIE, but many children are admitted to the hospital > 6 h, particularly in developing areas. We aimed to determine whether hypothermia treatment could remain effective within 12 h after birth.

**Methods:**

According to their admission, 152 newborns were enrolled in the < 6 h and 6–12 h after HIE groups. All newborns received conventional treatment combined with mild head hypothermia therapy, according to our routine clinical practice. Some newborns only received conventional treatment (lacking informed consent). All newborns received amplitude-integrated electroencephalography (aEEG) monitoring for 4 h and neuron-specific enolase (NSE) measurement before and after 3 days of therapy.

**Results:**

Compared to the conventional treatment, hypothermia significantly improved the aEEG scores and NSE values in all newborns of the < 6-h group. In the 6–12-h group, the aEEG scores (F = 5.67, *P* < 0.05) and NSE values (F = 4.98, *P* < 0.05) were only improved in newborns with moderate HIE. Hypothermia treatment seems to have no effect in newborns with severe HIE after 6 h (*P* > 0.05). Hypothermia improved the rates of neonatal death and 18-month disability (all *P* < 0.01).

**Conclusions:**

In newborns with moderate HIE, starting hypothermia therapy < 6 h and 6–12 h after HIE showed curative effects. In those with severe HIE, only starting hypothermia therapy within 6 h showed curative effects.

## Background

Neonatal hypoxic-ischemic encephalopathy (HIE) remains a devastating cause of death in the perinatal period as well as of future neurodevelopmental abnormalities [1, 2]. Hypothermia is a proven effective treatment of HIE and can improve survival and long-term prognosis of children [3, 4]. It has been suggested that hypothermia treatment of HIE should start within 6 h after hypoxia ischemia [5], but many newborns are admitted to the hospital > 6 h after HIE, particularly those living in rural areas. In addition, a number of factors can lead to delays in treatment initiation; parents are sometimes unsure of symptoms and may take some time before going to the hospital, or the hypothermia devices may be broken or unavailable. In patients admitted > 6 h after HIE, hypothermia can still be carried out and provide some benefits.

The protocols actually being used are mainly based on animal data. Indeed, the 6-h limit for hypothermia initiation comes from data suggesting that the effectiveness of hypothermia diminishes as time increases from the hypoxic ischemic event, with the closing of the therapeutic window occurring 5.5–8 h after the event [6]. Nevertheless, the exact timing of the therapeutic window after HIE is mostly unknown in human newborns and needs to be further investigated [7].

Amplitude-integrated electroencephalography (aEEG) reveals the changes of brain physiology and identifies subclinical seizures in the early stage of brain hypoxic-ischemia. Indeed, aEEG has high sensitivity, specificity, and prognostic value in brain function monitoring. Neuron specific enolase (NSE) is an early biochemical index for neonatal brain damage, and can also help determine the degree of neuronal damage and evaluate prognosis.

This study aimed to assess newborns with HIE by observing aEEG changes and NSE levels before and after hypothermia treatment, in order to explore the clinical curative effect of different initiating times for mild hypothermia treatment of HIE.

## Methods

### Patients and grouping

Newborns with HIE were enrolled between August 2013 and August 2014 at the Neonate Department of our hospital [8]. The inclusion criteria were: 1) gestational age ≥ 36 weeks; 2) birth weight ≥ 2500 g; and 3) admission within 12 h of birth. The exclusion criteria were: 1) major congenital abnormalities; 2) known or suspected chromosomal abnormalities; 3) major brain malformations; or 4) aEEG abnormalities from causes other than HIE.

The diagnostic criteria for HIE were: 1) evidence of moderate or severe clinical encephalopathy in the first 12 h of life; and 2) evidence of fetal distress, with at least one of the following: a) Apgar score ≤ 5 at 5 min; b) continued need for ventilation initiated at birth and for at least 10 min; and/or c) pH ≤ 7.00 in arterial cord blood or other sample in the first hour of life [3, 4]. Encephalopathy was classified as mild, moderate or severe according to a previously reported scale that focuses on the level of alertness [9, 10].

The study was approved by the institutional ethics committee. Informed consent was obtained from the legal guardians.

The patients were divided into the mild hypothermia and control groups according to the decision of the parents. According to the treatment starting time, they were further divided into the < 6 h and 6–12 h groups. According to HIE severity, they were divided into the moderate and severe groups.

### Therapies

The study enrolled all newborns with HIE undergoing the neonatal HIE treatment plan defined by the “Five-year Research Project HIE Cooperative Group” [11]. The control group included the newborns who received conventional treatment. The mild hypothermia group included the newborns who received conventional treatment combined with mild head hypothermia therapy after admission. For mild hypothermia, the Olympic Cool-Cap 004204 ice Cap system was used. The aim of hypothermia was to achieve rectal temperature of 34–35 °C, anterior fontanelle temperature of 20–25 °C, and skin temperature of 33–34.5 °C. The instrument entered the rewarming process automatically after 72 h of treatment. Table temperature was adjusted according to the anal temperature + 0.5 °C, based on computer interface prompts [12].

### aEEG monitoring

aEEG was performed using the NicoletOne 32-lead brain function monitoring instrument (Olympic company, USA) and according to the international 10/20 standard electrode placement system, with C3-C4 and P3-P4 in bilateral central and parietal regions, respectively, as lead signal patch; F3-F4 was used as the reference electrode [13]. Particular care was taken to place the electrodes and to be sure that they made contact with the skin. All newborns were immediately placed in a natural quiet environment upon admission. aEEG monitoring was carried out for 4 h and repeated after 3 days of treatment.

### aEEG interpretation

A synthetic marking system established by Burdjalov was used to analyze the graphs for continuity, periodicity, continuous voltage, lower boundary values, and narrow-band widths of aEEG [14]. The specific criteria are shown in Table 1.

**Table 1:** Scoring system of amplitude integrated electroencephalography (aEEG)

Score	Curve variability	SWC	Lower boundary values	Narrow-band width and lower boundary voltage
0	No variation curve	None	Seriously inhibited (< 3 μV)	Amplitude suppression: low amplitude (≤15 μV) and low voltage (5 μV)
1	Some curve, no sinusoidal ariation	Starting occurrence period	Some inhibition (3~ 5 μV)	Immaturity amplitude: high amplitude (> 20 μV) or medium amplitude (15~ 20 μV) and low voltage (5 μV)
2	Sinusoidal ariation	No clear	No inhibition (> 5 μV)	Immaturity amplitude: high amplitude (> 20 μV) and high voltage (> 5 μV)
3		Clear period, but suspension		Gradually mature amplitude: medium amplitude (15~ 20 μV) and high voltage (> 5 μV)
4		Clear period, no suspension		Mature amplitude: low amplitude (< 15 μV) and high voltage (> 5 μV)
5		Rule and mature period		

### NSE measurements

Venous blood (1 ml) was collected upon admission and after three days of treatment. Serum was prepared routinely. A commercial enzyme-linked immunoassay (ELISA) was used to measure NSE (North Institute of Biotechnology Products, Beijing, China), according to the manufacturer’s instructions. The serum samples were tested as soon as possible after preparation.

### Follow-up

Magnetic resonance imaging (MRI) was performed 2 weeks after HIE. The results were recorded as normal vs. abnormal. The rates of severe disability and death at 18 months were calculated.

### Statistical analysis

SAS 9.1 (SAS Institute, Cary, NY, USA) was used for all analyses. Categorical data were assessed using the Chi-square test. Continuous variables were analyzed using one way analysis of variance and the Tukey’s post hoc test. *P* < 0.05 was considered statistically significant.

## Results

### Baseline characteristics of the patients

A total of 2988 newborns were treated at our Neonatology Department during the study period. Among them, 513 had HIE, and 152 cases were enrolled based on the eligibility criteria. The mild hypothermia group included 63 newborns (< 6 h subgroup, 35 cases; 6–12 h subgroup, 28 cases). There were 89 newborns in the control group (< 6 h subgroup, 48 cases; 6–12 h group, 41 cases). The characteristics of the newborns are shown in Table 2. There were no significant differences among the subgroups for gestational age, birth weight, gender, delivery mode, 5-min Apgar score, HIE stage, and aEEG score. Due to changes of NSE levels with time after hypoxia, the values in different time windows within a given group changed; nevertheless, in both groups, similar NSE values were obtained for the same initiating window.

**Table 2:** General characteristics of the patients

	Control		Hypothermia		*P*
	< 6 h	≧6 h	< 6 h	≧6 h	
n	48	41	35	28	
Gestational age	38.5 ± 1.4	40 ± 0.8	38.4 ± 1.47	38.1 ± 0.9	0.16
Birth weight (kg)	3.44 ± 0.68	3.13 ± 0.34	3.17 ± 0.42	3.27 ± 0.34	0.15
Gender (male)	30 (62%)	26 (63%)	20 (57%)	23 (82%)	0.19
Cesarean section	18 (37%)	15 (36%)	15 (42%)	8 (28%)	0.71
5-min Apgar grade < 7	34 (50%)	30 (53%)	16 (46%)	13 (46%)	0.35
HIE					
Mild	15 (31%)	17 (41%)	18 (51%)	14 (50%)	
Moderate	18 (38%)	12 (29%)	11 (31%)	9 (32%)	0.49
Severe	15 (31%)	12 (29%)	6 (17%)	5 (18%)	
aEEG marking	3.6 ± 3.1	3.8 ± 3.0	4.6 ± 2.5	4.5 ± 3.1	0.45
NSE value	25.6 ± 10.9	38.1 ± 24.6	29.5 ± 27	41 ± 25.6	< 0.01

*HIE* Hypoxia ischemic encephalopathy, *aEEG* Amplitude integrated electroencephalography, *NSE* Neuron-specific enolase

### aEEG values and NSE levels in the hypothermia and control groups

**Fig. 1:**
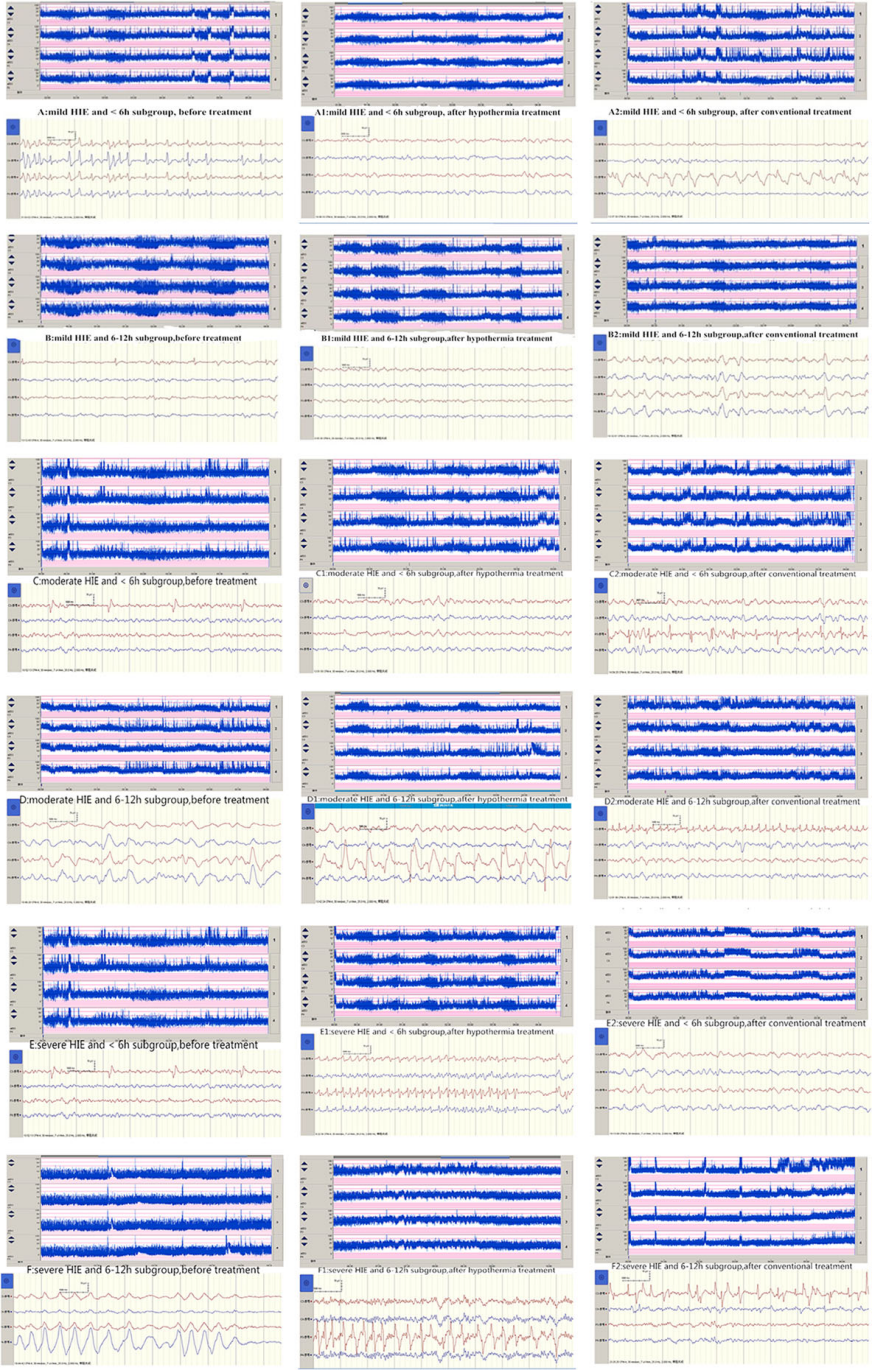
aEEG image comparison at all levels in different time windows of hypothermia treatment

There were no significant differences in aEEG values and NSE levels between the hypothermia and control groups, both for the < 6 h and 6–12 h subgroups, in newborns with mild HIE after 3 days of treatment (*P* > 0.05) (Fig. 1). The hypothermia and control groups with treatment starting time < 6 h in patients with moderate and severe HIE showed statistically significant differences in aEEG scores (Table 3) and NSE levels (Table 4) after 3 days of treatment (*P* < 0.05). When treatment started at 6–12 h, aEEG scores and NSE levels were significantly different after 3 days only in patients with moderate HIE (*P* < 0.05). Newborns with severe HIE showed no significant difference between the two groups after treatment (*P* > 0.05) (Tables 3 and 4).

**Table 3:** Changes in aEEG scores after 3 days of treatment in children with HIE

∆aEEG							
Group	Time window	Mild		Moderate		Severe	
		N	Mean ± SD	N	Mean ± SD	N	Mean ± SD
Control group	< 6 h	13	3.6 ± 2.2	15	4.7 ± 1.7^b1^	13	3.0 ± 2.1^c1^
	≥ 6 h	13	3.7 ± 2.2	10	3.7 ± 3.4^b2^	11	2.1 ± 1.4^c2^
Hypothermia group	< 6 h	14	4.5 ± 2.3	9	1.8 ± 0.7^b1^	5	0.2 ± 0.4^c1^
	≥ 6 h	11	4.1 ± 2.7	8	2.1 ± 1.0^b2^	5	1.0 ± 0.7^c2^
*P*		0.76^a^		< 0.005^b^		< 0.05^c^	

∆ Before and after treatment; ^a^no statistically significant difference between mild HIE groups (*P* > 0.05), ^b^moderate HIE groups showed statistically significant differences (*P* < 0.01): ^b1^moderate HIE and < 6 h subgroup (*P* < 0.05), ^b2^ moderate HIE and 6-12 h subgroup (*P* < 0.05); ^c^ severe HIE patients showed statistically significant differences between the subgroups (*P* < 0.05): ^c1^severe HIE and < 6 h subgroup (*P* < 0.05) ^c2^severe HIE and 6-12 h subgroup (*P* > 0.05); two initiation time windows in the hypothermia group showed no difference in moderate HIE patients (^b1b2^ HIE; *P* > 0.05) but in severe HIE patients (^c1c2^ *P* < 0.05)

**Table 4:** Changes in NSE levels after 3 days of treatment in children with HIE

∆NSE							
Group	Time window	Mild		Moderate		Severe	
		N	Mean ± SD	N	Mean ± SD	N	Mean ± SD
Control group	< 6 h	14	4.0 ± 0.6	14	19.1 ± 2.4^b1^	13	12.1 ± 0.3^c1^
	≥ 6 h	13	5.2 ± 1.5	9	21.4 ± 3.6^b2^	9	16.8 ± 5.2^c2^
Hypothermia group	< 6 h	15	2.0 ± 0.8	10	13.7 ± 1.8^b1^	6	14.3 ± 3.9^c1^
	≥ 6 h	9	3.9 ± 2.8	7	15.3 ± 5.1^b2^	5	19.8 ± 1.9^c2^
*P*		0.35^a^		< 0.01^b^		< 0.05^c^	

∆ Before and after treatment; ^a^no statistically significant difference between mild HIE groups (*P* > 0.05), ^b^moderate HIE groups showed statistically significant differences (*P* < 0.01): ^b1^moderate HIE and < 6 h subgroup (*P* < 0.05), ^b2^ moderate HIE and 6-12 h subgroup (*P* < 0.05); ^c^severe HIE patients showed statistically significant differences between the subgroups (*P* < 0.05): ^c1^severe HIE and < 6 h subgroup (*P* < 0.05) ^c2^severe HIE and 6-12 h subgroup (*P* > 0.05); two initiation time windows in the hypothermia group showed no difference in moderate HIE patients (^b1b2^ HIE; *P* > 0.05) but in severe HIE patients (^c1c2^ *P* < 0.05)

### Mid- and long-term outcomes

Table 5 presents the results of the 2-week MRI, as well as the 18-month outcomes. The rate of normal MRI was higher in the hypothermia group compared with the control group (*P* < 0.01). In the hypothermia group, the rate of normal MRI results was higher in the < 6 h subgroup (*P* < 0.01). The 18-month rate of severe disability and the rate of neonatal death were lower in the hypothermia group compared with the control group (both *P* < 0.01). There were no differences between the < 6 h and 6–12 h groups.

**Table 5:** Results of 2-week MRI, 18-month disability rate, and neonatal death

	Controls		Hypothermia		*P*
	< 6 h	6–12 h	< 6 h	6–12 h	
n	48	41	35	28	
MRI during the second week					
Normal	26 (54.2%)	23 (56.1%)	30 (85.7%)	20 (71.4%)	< 0.01
Abnormal	22 (45.8%)	18 (43.9%)	15 (14.3%)	8 (29.6%)	< 0.01
Severe disability	19 (39.6%)	16 (39.0%)	9 (25.7%)	7 (28.6%)	< 0.01
Neonatal death	4 (8.3%)	3 (7.3%)	1 (2.9%)	1 (3.6%)	< 0.01

## Discussion

It has been suggested that mild hypothermia treatment of HIE should occur at < 6 h after HIE, but many newborns are admitted to the hospital > 6 h, particularly in developing areas. Therefore, this study aimed to determine whether hypothermia treatment could remain effective within 12 h after birth. The results showed that in newborns with moderate HIE, both < 6 h and 6–12 h hypothermia therapy starting times showed curative effects. In those with severe HIE, only starting hypothermia therapy within 6 h showed curative effects.

The etiology of neonatal HIE is complex [15]. It begins with cerebral flow reperfusion at 6–24 h after several hours or days of hypoxia ischemia, leading to mitochondrial oxidative damage and neuronal energy failure. Excess free radicals, intracellular Ca^2+^ overload, and large amounts of excitatory amino acids, combined with the action of inflammatory cells and inflammatory cytokines, will lead to cell death. More important hypoxia severity and duration will lead to greater pathological changes. The key link is secondary energy failure, which activates a series of biochemical reactions, finally causing or aggravating neuronal death. Secondary energy failure after 6–12 h offers a time window for disease development. Multicenter studies [16–18] also indicated that hypothermia treatment can significantly improve the prognosis of newborns with moderately severe HIE, as well as the time window of treatment < 6 h after birth. Nevertheless, some authors proposed that delaying treatment to within 10 h after hypoxia ischemia results in similar effectiveness [19], but studies reporting a treatment delay of 10 or even 12 h are scarce. Meanwhile, quite a few newborns are admitted to the hospital more than 6 h after hypoxic ischemia. Specifically, Western China has a relatively underdeveloped medical and transportation systems. The patients could benefit from hypothermia if treatment start could be delayed to > 6 h after birth. Therefore, this study included newborns with hypoxic ischemic brain damage admitted 6–12 h after birth.

Few studies have assessed hypothermia in the treatment of mild HIE. Zhou et al. [20] suggested that newborns with mild HIE have less neurological sequelae, and that the curative effect of hypothermia in such patients is not significant. Some studies found that newborns with mild HIE exhibit poor cognitive function in childhood [21], with lower memory score compared with the normal group [22]. In the present study, hypothermia improved the rates of neonatal death and 18-month disability. The time window of < 6 h led to better rates of 18-month disability, but without difference on death. Therefore, newborns with HIE could benefit from hypothermia treatment, but further studies with larger sample size are needed. In particular, it is difficult to evaluate the severity of HIE according to objective indexes early after birth, and only treating newborns with moderately severe HIE with hypothermia may be leaving out newborns that could be helped. In this study, newborns with mild HIE underwent hypothermia treatment for 72 h after informed consent was provided by their legal guardians. The findings suggest that delayed hypothermia therapy for newborns with HIE is effective, although further clinical studies are needed for confirmation. If hypothermia treatment is delayed by more than 6 h after birth, the best treatment window of HIE patients might be missed.

aEEG can sensitively reflect the change of brain physiology at the early stage of brain ischemic hypoxia. This is the only way to detect subclinical seizures and reversible changes of brain function [23, 24]. Some authors [25] indicated that aEEG can confirm encephalopathy within 6 h after HIE. Indeed, aEEG could predict abnormal neurological development with 82% specificity, 85% positive rate, and 100% negative rate. In the present study, aEEG was an effective method of monitoring the newborns since some improvements were seen in patients receiving hypothermia.

NSE is a soluble protein and exists in central nerve cells and peripheral nerve tissues, more specifically in neuroendocrine cells. It is very rare in normal body fluids (including blood and the cerebrospinal fluid). In case of neuronal injury and necrosis, the blood brain barrier is damaged [26]. Celtik et al. [27] demonstrated that NSE can be used as a quantitative marker of brain damage. This study revealed that NSE levels in newborns with moderately severe HIE were higher than in those with mild HIE before treatment. In addition, NSE levels were positively associated with the degree of brain damage. In HIE newborns and within 48 h, plasma NSE levels are significantly higher than control values, and even more significantly elevated in severe HIE, indicating that hypoxic ischemia and reperfusion injury is an important mechanism of HIE [28]. NSE levels are associated with disease course and with serious brain edema. It has been shown that NSE levels peak at 24 h after hypoxic brain damage [29]. Therefore, the time of specific peak in the disease course may still need to be assessed in studies with a large sample size.

Mild-to-moderate brain edema is reversible; therefore, patients with mild HIE, whose brain damage is still mostly at the state of brain edema, can be fully restored a week or so, even with routine treatment. As shown above, no difference in the curative effect of hypothermia treatment was found in newborns with mild HIE between the < 6 h and 6–12 h groups. In addition, these patients showed no differences compared with the control group, suggesting a good prognosis in newborns with mild HIE, even without hypothermia treatment. Nevertheless, long-term prognosis assessment and long-term follow-up are needed to investigate whether newborns with mild HIE would show long-term developmental issues such as cognitive dysfunction. This would determine whether such patients need hypothermia treatment at all.

A remarkable curative effect was obtained in newborns with moderate HIE for both time windows (< 6 h and 6–12 h) of hypothermia treatment, in disagreement with some reports [30]. In these patients, the series of biochemical reaction leading to serious hypoxic brain damage might not have started. Even after a long period of time, deterioration can still be prevented to a certain degree through effective treatment.

Severe brain edema entering the stage of neuron necrosis is irreversible. The cerebral blood flow decreases significantly within 12 h after severe asphyxia, and gradually increases by 24–120 h. By then, the brain is in a state of multiple perfusion, and has launched a series of biochemical reactions, with subsequent nerve cell necrosis and apoptosis. For severe hypoxic brain damage, even if the corresponding hypothermia treatment takes place and reduces the cerebral metabolic process beyond a certain time window, important neurons have already been involved in irreversible necrosis or even infarction. This explains the poor curative effect. Our findings corroborate numerous studies showing that hypothermia treatment of newborns with severe HIE at < 6 h after birth is useless [31].

This study is not without limitations. The sample size was small and from a single center. In addition, due to the principles of informed consent by family members regarding hypothermia treatment and because of the economic status and education background of the families, randomization was impossible. Finally, no follow-up was performed to examine the impact of treatment on long-term cognitive functions.

## Conclusions

In newborns with moderate HIE, both time windows (< 6 h and 6–12 h) for starting hypothermia treatment showed curative effects. The treatment effects were better when the treatment was started early. Meanwhile, newborns with severe HIE only showed a therapeutic effect for hypothermia treatment beginning within 6 h. Good prognosis was obtained in newborns with mild HIE, even without hypothermia treatment. Whether newborns with mild HIE would show long-term developmental issues (such as cognitive dysfunction) needs to be assessed in long-term follow-up.

## Abbreviations

aEEG: Amplitude integrated electroencephalography
ELISA: Enzyme-linked immunoassay
HIE: Neonatal hypoxic-ischemic encephalopathy
NSE: Neuron specific enolase
